# Draft Genome Sequence of *Kingella negevensis* SW7208426, the First European Strain of *K. negevensis* Isolated from a Healthy Child in Switzerland

**DOI:** 10.1128/genomeA.00571-17

**Published:** 2017-06-29

**Authors:** Nawal El Houmami, Jacques Schrenzel, Pablo Yagupsky, Catherine Robert, Dimitri Ceroni, Didier Raoult, Pierre-Edouard Fournier

**Affiliations:** a Aix-Marseille University, URMITE, UM63, CNRS 7278, IRD 198, Inserm 1095, Institut Hospitalo-Universitaire Méditerranée Infection, AP-HM Public Hospitals, Marseille, France; b Bacteriology and Genomic Research Laboratories, Geneva University Hospitals and Geneva University, Geneva, Switzerland; c Clinical Microbiology Laboratory, Soroka University Medical Center, Beer-Sheva, Israel; d Service d’Orthopédie Pédiatrique, Département de l’Enfant et de l’Adolescent, Hôpitaux Universitaires de Genève, Geneva, Switzerland

## Abstract

We report here the draft genome of *Kingella negevensis* strain SW7208426, isolated from the oropharynx of a healthy 6-year-old boy in Geneva, Switzerland. To our knowledge, this is the first genome report of the newly described *K. negevensis* species from Europe.

## GENOME ANNOUNCEMENT

*Kingella negevensis* is a newly described Gram-negative bacterium belonging to the genus *Kingella* in the *Neisseriaceae* family (1). Asymptomatically carried in the oropharynx of young children, most *K. negevensis* strains have been isolated in southern Israel to date (1). To the best of our knowledge, no genome sequence from European strains of this novel *Kingella* sp. is available in international databases. Herein, we briefly present the genome sequence of *K. negevensis* strain SW7208426, which was isolated from the oropharynx of a healthy 6-year-old boy at the Hôpital des Enfants in Geneva, Switzerland. The study received institutional review board approval (09-029R, Mat-Ped 09-008R).

Using genotyping by pulsed-field gel electrophoresis and 16S rRNA typing, strain SW7208426 was classified as belonging to genotype η, which is the only of the six originally reported *K. negevensis* genotypes to have been detected in the United States as well (1). Strain SW7208426 was subcultured in blood agar medium at 37°C in a 5% CO_2_-enriched atmosphere. Genomic DNA was extracted as previously described (1) and then sequenced by using an Illumina MiSeq sequencer (Illumina Inc, San Diego, CA, USA) and the mate-pair strategy with the Nextera Mate Pair Sample prep kit (Illumina). The genome assembly was performed with a pipeline utilizing different software—namely, Velvet (2), Spades (3), and SOAPdenovo2 (4)—on trimmed (MiSeq and Trimmomatic [5] software) or untrimmed data (only MiSeq software). For each of the six assemblies performed, GapCloser (4) was used to reduce gaps. This yielded a draft genome consisting of 12 scaffolds (50 contigs) for a total of 2,187,490 bp and a G+C content of 45.27%. Open reading frames (ORFs) were predicted using Prodigal (6) with default parameters and the predicted bacterial protein sequences were searched against the Clusters of Orthologous Groups (COG) database using BLASTp. The tRNAScanSE (7) tool was used to find tRNA genes, whereas rRNA genes were found using RNAmmer (8). Lipoprotein signal peptides and the number of transmembrane helices were predicted using Phobius (9). ORFans were identified if the BLASTp search did not give positive results (*E* value smaller than 1e^−03^ for ORFs with a sequence size superior to 80 amino acids [aa] or *E* value smaller than 1e^−05^ for ORFs with sequence length smaller than 80 aa). The genome was shown to encode 65 predicted RNAs, including 4 complete rRNA operons and 53 tRNAs. Of the 2,253 predicted genes, a total of 1,607 genes (73.25%) were assigned a putative function; 75 genes were identified as ORFans (3.42%); and 36 genes (19.87%) were annotated as coding hypothetical proteins. Further genomic analyses are required in order to elucidate the molecular characteristics involved in the human carriage and dissemination of *K. negevensis* worldwide. Strain SW7208426 is deposited in the CSUR collection under number P3485.

### Accession number(s).

This whole-genome shotgun project has been deposited in GenBank under the accession number FXBH00000000. The version described in this paper is the first version, FXBH01000000.
